# Prevalence and determinants of self-reported high blood pressure among women of reproductive age in Benin: a population-based study

**DOI:** 10.1186/s40885-020-00145-z

**Published:** 2020-07-01

**Authors:** Michael Ekholuenetale, Amadou Barrow

**Affiliations:** 1 Department of Epidemiology and Medical Statistics, Faculty of Public Health, College of Medicine, University of Ibadan, Ibadan, Nigeria; 2 Department of Public & Environmental Health, School of Medicine & Allied Health Sciences, University of The Gambia, Kanifing, The Gambia

**Keywords:** Hypertension, Noncommunicable diseases, Chronic diseases, Sub-Saharan Africa

## Abstract

**Background:**

Addressing chronic diseases is a challenge for healthcare systems worldwide, which have largely developed to deal with acute episodic care, rather than to provide organized care for people with age-long conditions. Therefore, exploring the prevalence and identifying the risk factors is a major approach to prevention and control of chronic diseases. The aim of this study was to examine the prevalence and factors associated with self-reported high blood pressure among women of reproductive age in Benin.

**Methods:**

We utilized population-based cross-sectional data from Benin Demographic and Health Survey (BDHS). BDHS 2017–18 is the round V of the survey. A total of 7712 women of reproductive age were included in this study. The outcome variable was self-reported high blood pressure. Percentages, chi-square test and multivariable logistic regression model were used to analyze the data. Results from the multivariable logistic model were presented as adjusted odds ratio (aOR) and confidence interval (95%CI). The significance level was set at *p* < 0.05.

**Results:**

The total prevalence of self-reported high blood pressure among women of reproductive age in The Gambia was about one-tenth (9.9%). Furthermore, geographical region was associated with high blood pressure. Women aged 45–49 years had increased odds of high blood pressure, when compared with women aged 15–19 years (aOR = 2.73; 95%CI: 1.10, 6.79). In addition, increased maternal enlightenment gave higher odds of high blood pressure, when compared to women with low maternal enlightenment (aOR = 1.41; 95%CI: 1.08, 1.84). Women with highest neighbourhood socioeconomic disadvantaged status (poor women) had 30% reduction in the odds of high blood pressure, when compared with women of low neighbourhood socioeconomic disadvantaged status (aOR = 0.70; 0.50, 0.99). Women having increased lifetime number of sex partners (total of 3 lifetime number of sex partners: aOR = 1.40; 95%CI: 1.01, 1.94; more than 3 total lifetime number of sex partners: aOR = 1.53; 95%CI: 1.01, 2.32) were more likely to have high blood pressure, when compared to women with only one lifetime number of sex partner.

**Conclusions:**

Emphasis on high blood pressure prevention methods and involvement of multiple sectors could help to disseminate health care interventions widely. Also, a concerted effort from the stakeholders in health care system and decision-makers is needed to address the drivers of high blood pressure while maintaining health system improvement strategies. The findings could prompt appropriate policy responses towards improving the knowledge and control of high blood pressure in Benin.

## Background

Globally, the burden of non-communicable diseases (NCDs) is worrisome due to the staggering number of deaths up to 41 million people, which is approximately 71% of all global deaths of 57 million in 2016 [1, 2]. Cardiovascular diseases account for majority of NCD deaths, or about 17.9 million (or 44% of all NCD deaths), followed by cancers (9.0 million or 22% of all NCD deaths), respiratory diseases including asthma and chronic obstructive pulmonary disease (3.8 million or 9% of all NCD deaths), and diabetes (1.6 million or 4% of all NCD deaths) [1, 2]. These 4 groups of diseases account for over 80% of all premature NCD deaths worldwide. NCDs continue to pose public health problems in several countries and responsible for the high number of deaths worldwide. The burden of these diseases is rising disproportionately among resource-poor settings. In 2016, over three-quarters of NCD deaths (31.5 million) occurred in resource-poor settings with about 46% of deaths occurring before the age of 70 years [1, 2].

Similar to many resource-poor settings in sub-Saharan Africa (SSA), Republic of Benin has a large burden of NCDs. The country has prevalence of deaths from NCDs at 37% accounting for a total number of NCD deaths of about 36,000, while the risk of premature death from target NCDs is 22% [1, 2]. The unabated burdens of poor health outcomes have largely contributed to the endorsement of the Sustainable Development Goals (SDGs) which characterize global efforts toward equity and equality in health care distribution, particularly the SDG-3. The focus of the goal is to help ensure healthy lives and promote well-being for all at all ages [3]. The fourth target of the SDG-3 (3.4) focuses on reducing premature mortality from NCDs by about one-third by 2030 through prevention and treatment [3]. It is remarkable that NCDs are progressively overriding health care needs in resource-poor settings and gaining in-depth policy recognitions. Adults in resource-poor settings faced higher risks (about 22%) approximately double the rate for adults in high-income countries (12%) [1].

NCDs have recently become a prominent cause of mortality, which accounts for nearly two-thirds of deaths worldwide, with more than three-quarters of NCD-related mortality occurring in resource-poor settings [4]. In SSA region, the problem of NCDs and their risk factors are on the increase with several causes. Consequently, healthy lifestyles and NCD control became the goals of Global Ministerial Conference, which prepared the platform for the United Nations (UN) Summit on NCD and subsequently, the declaration later in the year 2011 [5]. During the Moscow Declaration on NCDs in 2011, that proceeded from the ministerial conference, encompassed a responsibility from governments to create public policies that give equitable health-supporting conditions that make communities, families and individuals to have healthy behaviours, as they give precedence to NCD prevention and control, maintaining other health care objectives and to reinforced the commitment of multiple sectors to address NCDs risk factors.

Effectively tackling NCDs and their key risk factors requires a detailed understanding of the current status and progress being made at the country level. Feasible and cost-effective interventions exist to reduce the burden and impact of NCDs now and in the future. NCDs share several risk factors such as tobacco use, consumption of alcohol, old age, increased BMI, area of residence, work stress, triglyceride levels, family history, physical inactivity, high total cholesterol level, high adiposity, poor dietary habits, ethnicity, marital status, sex, salt use, less consumption of vegetable or fruit, increased fasting glucose, household wealth quintile, education, employment, sugary drinks intake, waist-to-hip ratio amongst others [6–11]. Prominently, high blood pressure (hypertension) has been reported as a key determinant of NCDs such as cardiovascular diseases [12]. Over the past four decades, the prevalence of hypertension has remained unabated. Approximately 600 million was estimated in 1980 and about 1 billion in 2010 due to population growth, lifestyle, and ageing [13]. Hypertension has remained a major risk factor for numerous cardiovascular diseases [14, 15]. Unfortunately, many people with hypertension are unaware of their condition [16]. In light of the above, the aim of this study was to examine the prevalence and factors associated with high blood pressure among women of reproductive age in Benin.

## Methods

### Data extraction

In this study, we utilized a population-based cross-sectional data from BDHS. BDHS 2017–18 is the V round of the survey. A total of 7712 women of reproductive age were included in this study. BDHS used a stratified multi-stage cluster random sampling technique. Data was collected on vital reproductive health issues via structured interviewer-administered questionnaires. DHS program was established by the United States Agency for International Development (USAID) in 1984. It was designed as a follow-up to the World Fertility Survey and the Contraceptive Prevalence Survey projects. It was first awarded in 1984 to Westinghouse Health Systems (which subsequently evolved into part of OCR Macro). The project has been implemented in overlapping five-year phases; DHS-I ran from 1984 to1990; DHS-II from 1988 to1993; and DHS-III from 1992 to1998. In 1997, DHS was folded into the new multi-project MEASURE program as MEASURE DHS+. Since 1984, more than 130 nationally representative household-based surveys have been completed under the DHS project in about 70 countries.

Many of the countries have conducted multiple DHS to establish trend data that enable them to gauge progress in their programs. Countries that participate in the DHS program are primarily countries that receive USAID assistance; however, several non-USAID supported countries have participated with funding from other donors such as UNICEF, UNFPA or the World Bank. DHS are designed to collect data on fertility and reproductive health, child health, family planning and HIV/AIDS. Due to the subject matter of the survey, women of reproductive age (15–49) are the main focus of the survey. Women eligible for an individual interview are identified through the households selected in the sample. Therefore, all DHS surveys utilize a minimum of two questionnaires-a Household Questionnaire and a Women’s Questionnaire. DHS data is publicly available and can be accessed from the MEASURE DHS database at http://dhsprogram.com/data/available-datasets.cfm. DHS are usually implemented by the National Population Commission (NPC) with financial and technical assistance by ICF International provisioned through the USAID-funded MEASURE DHS program. DHS involved multi-stage stratified cluster design based on a list of enumeration areas (EAs), which are systematically selected units from localities and constitute the Local Government Areas (LGAs). Details of the sampling procedure have been reported previously [17].

### Study area

There are twelve [12] geographical regions in Benin, namely; Alibori, Atacora, Atlantique, Borgou, Collines, Couffo, Donga, Littoral, Mono, Quémé, Plateau and Zou. The country spans from north to south and indeed a long stretched country in West Africa, which is located west of Nigeria and east of Togo, it is bordered to the north by Niger and Burkina Faso, in south by the Bight of Benin, in the Gulf of Guinea, that part of the tropical North Atlantic Ocean which is roughly south of West Africa. Benin’s coastline is approximately 121 km long, with an area of 112,622 km^2^. Benin has a population of approximately 10 million people (in 2013), Porto-Novo, a port on an inlet of the Gulf of Guinea is the nation’s capital city, largest city and economic capital is Cotonou. Spoken languages are French (official), Fon and Yoruba [18].

### Measurement of outcome variable

The outcome variable was measured dichotomously (yes vs. no) as reported by the respondents; “ever told has high blood pressure”.

### Independent variables

Household wealth quintile**:** principal components analysis (PCA) was used to assign the wealth indicator weights. This procedure assigned scores and standardized the wealth indicator variables such as; bicycle, motorcycle/scooter, car/truck, main floor material, main wall material, main roof material, sanitation facilities, water source, radio, television, electricity, refrigerator, cooking fuel, furniture, number of persons per room. The factor coefficient scores (factor loadings) and z-scores were calculated. For each household, the indicator values were multiplied by the loadings and summed to produce the household’s wealth index value. The standardized z-score was used to disentangle the overall assigned scores to the poorest/poorer/middle/richer/richest categories [19, 20]; Furthermore, neighbourhood socioeconomic disadvantaged level: used items such as; percentage women in rural residence, percentage women from poorest household wealth level, percentage women with no formal education and percentage women not working. Using PCA, factor coefficient scores and z-scores were calculated. Neighbourhood socioeconomic disadvantage score was standardised, with Z scores for individual neighbourhoods ranging from − 1.98 (relative advantage) to 1.69 (relative disadvantage). The standardized z-score was used to disentangle the overall assigned scores to low, medium and high socioeconomic disadvantaged levels. Women’s enlightenment level: percentage women with formal education, percentage women who read newspaper/magazines, percentage women who listen to radio and watch television; decision-making power: respondent involved in the decision on how to spend her earnings, respondent involved in the decision on her health care, respondent involved in the decision on large household purchases, respondent involved in the decision on visits to family or relatives, respondent involved in the decision on what to do with money husband/partner earns.

In addition, maternal age: 15–19/20–24/25–29/30–34/35–39/40–44/45–49; age at sexual debut: < 14 years/14–17 years/> 17 years/not had sex;number of children ever born: 1–2/3–4/> 4/no bith; religion: Christianity/Islam/Traditional and others; health insurance coverage: not covered/covered; maternal education: no formal education/primary/secondary/tertiary; place of residence: urban/rural; distance from health facility (km): 1/2/3/> 3; lifetime number of sex partners: 1/2/3/> 3; use of cigarettes or tobacco products: use/not use; ever used any method to delay pregnancy: yes/no; frequency of listening to radio/watching television: not at all/less than once a week/at least once a week; participation in labour force: working/not working; geographical region: Alibori, Atacora, Atlantique, Borgou, Collines, Couffo, Donga, Littoral, Mono, Quémé, Plateau and Zou were examined in this study. These factors were included based on previous studies that examined the factors associated with hypertension and related conditions [6–11].

### Ethical consideration

This study was based on the analysis of population-based dataset available in the public domain/online with all identifiers removed. The authors communicated with MEASURE DHS/ICF International and permission was granted to download and use the data. The DHS project obtained the required ethical approvals from the relevant research ethics committee in Benin, West Africa before the survey was conducted to ensure that the protocols are in compliance with the U.S. Department of Health and Human Services regulations for the protection of human subjects. Written informed consents were obtained from participants before being allowed in the surveys.

### Data analysis

We used the built-in survey command of Stata for all analyses to account for the sampling strata, primary sampling unit, and sampling weight provided in the dataset. Prevalence of high blood pressure was reported in percentage. Correlation matrix was used to conduct multicollinearity diagnostics to examine the interdependence between explanatory variables using a cut-off minimum of 0.7 known to cause concerns in multicollinearity [21]. Furthermore, women’s enlightenment was retained in place of media use (radio and television) and maternal education, while neighbourhood socioeconomic disadvantaged status was retained in place of household wealth index, residence and maternal education due to collinearity. Furthermore, variables that were statistically significant in the Chi-squared bivariate analysis were added in the multivariable regression logistic models to examine the factors associated with high blood pressure. The level of statistical significance was set at 5%. All data analyses were conducted using Stata 14.0 (Statacorp, College Station, Texas, United States of America).

## Results

The prevalence of high blood pressure among women aged 15–49 years in Benin, West Africa was about one-tenth (9.9%). The distribution of high blood pressure by maternal characteristics was presented in Table 1. The prevalence of high blood pressure increased as a woman advances in age such that women aged 45–49 years had highest prevalence of high blood pressure (19.5%). The magnitude of high blood pressure was higher in urban residence (11.6%) and Oueme geographical region (19.5%). High blood pressure was highest among the well-off, such that women from the richest household wealth status reported about 14.6%. Women with tertiary education (16.0%), working/employed (11.3%), Christians (11.8%), lowest neighbourhood socioeconomic disadvantaged (13.3%), covered by health insurance (19.1%), ever used contraceptive (13.2%), have over 3 lifetime sex partners (14.1%) reported increased prevalence of high blood pressure respectively.

**Table 1:** Percentages and factors associated with self-reported high blood pressure among women of reproductive age in Benin (*n* = 7712)

Variable	Number of women (%)	Number of women with high blood pressure	Percentage (%) of women with high blood pressure	Unadjusted OR (95%CI)	Adjusted OR (95%CI)
**Maternal age**					
15–19	1623 (21.1)	41	2.5	1.00	1.00
20–24	1422 (18.4)	88	6.2	2.54 (1.74–3.71)*	0.82 (0.34–1.96)
25–29	1488 (19.3)	137	9.2	3.91 (2.74–5.59)*	0.95 (0.40–2.23)
30–34	1029 (13.3)	113	11.0	4.76 (3.30–6.87)*	1.21 (0.50–2.91)
35–39	942 (12.2)	138	14.6	6.62 (4.63–9.48)*	1.46 (0.60–3.56)
40–44	613 (8.0)	118	19.3	9.20 (6.36–13.31)*	2.22 (0.90–5.50)
45–49	595 (7.7)	116	19.5	9.34 (6.45–13.53)*	2.73 (1.10–6.79)*
**Place of residence**					
Urban	3431 (44.5)	397	11.6	1.00	
Rural	4281 (55.5)	354	8.3	0.69 (0.59–0.80)*	
**Geographical region**					
Alibori	861 (11.2)	50	5.8	1.00	1.00
Atacora	666 (8.6)	15	2.3	0.37 (0.21–0.67)*	0.23 (0.08–0.69)*
Atlantic	808 (10.5)	108	13.4	2.50 (1.76–3.55)*	0.80 (0.43–1.47)
Borgou	858 (11.1)	26	3.0	0.51 (0.31–0.82)*	0.42 (0.22–0.78)*
Collines	716 (9.3)	52	7.3	1.27 (0.85–1.90)	0.89 (0.50–1.58)
Couffo	493 (6.4)	74	15.0	2.86 (1.96–4.18)*	2.02 (1.09–3.73)*
Donga	451 (5.9)	16	3.6	0.60 (0.34–1.06)	0.25 (0.09–0.67)*
Littoral	661 (8.6)	107	16.2	3.13 (2.20–4.46)*	1.65 (0.89–3.05)
Mono	398 (5.2)	30	7.5	1.32 (0.83–2.11)	0.79 (0.40–1.53)
Oueme	616 (8.0)	120	19.5	3.92 (2.77–5.56)*	3.14 (1.80–5.47)*
Plateau	465 (6.0)	76	16.3	3.17 (2.17–4.62)*	2.05 (1.19–3.50)*
Zou	719 (9.3)	77	10.7	1.95 (1.34–2.82)*	1.29 (0.73–2.27)
**Wealth index**					
Poorest	1468 (19.0)	77	5.3	1.00	
Poorer	1528 (19.8)	112	7.3	1.43 (1.06–1.93)*	
Middle	1359 (17.6)	115	8.5	1.67 (1.24–2.25)*	
Richer	1538 (19.9)	181	11.8	2.41 (1.82–3.18)*	
Richest	1819 (23.6)	266	14.6	3.09 (2.38–4.03)*	
**Maternal education**					
No formal education	4236 (54.9)	405	9.6	1.00	
Primary	1516 (19.7)	172	11.4	1.21 (1.01–1.46)*	
Secondary	1773 (23.0)	144	8.1	0.84 (0.69–1.02)	
Tertiary	187 (2.4)	30	16.0	1.81 (1.21–2.71)*	
**Participation in labour force**					
Working	5833 (75.6)	656	11.3	2.38 (1.91–2.97)*	0.72 (0.34–1.49)
Not working	1879 (24.4)	95	5.1	1.00	1.00
**Religion**					
Christianity	4230 (54.9)	497	11.8	1.00	1.00
Islam	2298 (29.8)	145	6.3	0.51 (0.42–0.61)*	1.07 (0.75–1.52)
Traditional and others	1184 (15.3)	109	9.2	0.76 (0.61–0.95)*	1.02 (0.74–1.40)
**Neighbourhood socioeconomic disadvantaged status**					
Tertile 1 (lowest)	2563 (33.2)	341	13.3	1.00	1.00
Tertile 2	2565 (33.3)	260	10.1	0.74 (0.62–0.87)*	0.89 (0.67–1.20)
Tertile 3 (highest)	2584 (33.5)	150	5.8	0.40 (0.33–0.49)*	0.70 (0.50–0.99)*
**Women’s enlightenment level**					
Low	2837 (36.8)	194	6.8	1.00	1.00
Moderate	3042 (39.4)	358	11.8	1.82 (1.51–2.18)*	1.41 (1.08–1.84)*
High	1833 (23.8)	199	10.9	1.66 (1.35–2.04)*	1.20 (0.84–1.72)
**Decision making power**					
Low	1358 (35.9)	157	11.6	1.00	1.00
Moderate	1751 (46.4)	277	15.8	1.44 (1.17–1.77)*	1.05 (0.81–1.36)
High	668 (17.7)	88	13.2	1.16 (0.88–1.53)	0.96 (0.68–1.35)
**Number of children ever born**					
1–2	1945 (25.2)	200	10.3	1.00	1.00
3–4	1691 (21.9)	200	11.8	1.17 (0.95–1.44)	0.99 (0.72–1.38)
> 4	2027 (26.3)	283	14.0	1.42 (1.17–1.72)*	0.97 (0.66–1.41)
No birth	2049 (26.6)	68	3.3	0.30 (0.23–0.40)*	1.17 (0.89–3.53)
**Covered by health insurance**					
Not covered	7628 (98.9)	735	9.6	1.00	1.00
Covered	84 (1.1)	16	19.1	2.21 (1.27–3.82)*	1.78 (0.89–3.53)
**Watch television**					
Not at all	4777 (61.9)	392	8.2	1.00	
Less than once a week	1338 (17.4)	160	12.0	1.52 (1.25–1.85)*	
At least once a week	1597 (20.7)	199	12.5	1.59 (1.33–1.91)*	
**Listen to radio**					
Not at all	3311 (42.9)	249	7.5	1.00	
Less than once a week	1648 (21.4)	161	9.8	1.33 (1.08–1.64)*	
At least once a week	2753 (35.7)	341	12.4	1.74 (1.46–2.06)*	
**Ever used any method to delay pregnancy**					
Yes	2371 (30.7)	312	13.2	1.69 (1.45–1.97)*	1.25 (0.99–1.58)
No	5341 (69.3)	439	8.2	1.00	1.00
**Usage of cigarettes or tobacco products**					
Use	216 (2.8)	8	3.7	1.00	1.00
Not use	7496 (97.2)	743	9.9	2.86 (1.41–5.82)*	1.21 (0.41–3.61)
**Age at first sex**					
< 14	525 (6.8)	39	7.4	1.00	1.00
14–17	3887 (50.4)	381	9.8	1.35 (0.96–1.91)	1.11 (0.69–1.77)
> 17	2364 (30.7)	319	13.5	1.94 (1.37–2.74)*	1.07 (0.65–1.75)
Not had sex	936 (12.1)	12	1.3	0.16 (0.08–0.31)*	–
**Total lifetime number of sex partners**					
1	3067 (45.5)	282	9.2	1.00	1.00
2	2121 (31.4)	244	11.5	1.28 (1.07–1.54)*	1.04 (0.80–1.34)
3	955 (14.2)	122	12.8	1.45 (1.15–1.81)*	1.40 (1.01–1.94)*
> 3	603 (8.9)	85	14.1	1.62 (1.25–2.10)*	1.53 (1.01–2.32)*
**Distance to health facilities (kilometer)**					
1	2400 (37.0)	233	9.7	1.00	1.00
2	1148 (17.7)	121	10.5	1.10 (0.87–1.38)	1.31 (0.97–1.77)
3	763 (11.7)	82	10.8	1.12 (0.86–1.46)	1.24 (0.87–1.77)
> 3	2184 (33.6)	169	7.7	0.78 (0.63–0.96)*	1.11 (0.84–1.48)

NB: Women’s enlightenment was retained in the adjusted logistic model in place of listening to radio, watching television and maternal education, while neighbourhood socioeconomic disadvantaged status was retained in the adjusted logistic model in place of household wealth index, place of residence and maternal education due to collinearity;

*significant at *p* < 0.05

Based on the results from multivariable logistic regression, several factors were found to be associated with high blood pressure among women of reproductive age. Notably, geographical region was associated with high blood pressure. In addition, women aged 45–49 years had increased odds of high blood pressure, when compared with women aged 15–19 years (aOR = 2.73; 95%CI: 1.10, 6.79). In addition, increased maternal enlightenment gave increased odds of high blood pressure, when compared to women with low maternal enlightenment (aOR = 1.41; 95%CI: 1.08, 1.84). Women with highest neighbourhood socioeconomic disadvantaged status (poor women) had 30% reduction in the odds of high blood pressure, when compared with women of low neighbourhood socioeconomic disadvantaged status (aOR = 0.70; 0.50, 0.99). Women having increased lifetime number of sex partners (total of 3 lifetime number of sex partners: aOR = 1.40; 95%CI: 1.01, 1.94; more than 3 total lifetime number of sex partners: aOR = 1.53; 95%CI: 1.01, 2.32) were more likely to have high blood pressure, when compared to women with only one lifetime number of sex partner. See Table 1 for the details.

## Discussion

This study has become the foremost to examine the prevalence and determinants of high blood pressure among women of reproductive age in Benin, using nationally representative data. High blood pressure was reported in about one-tenth of the women. In previous studies including a systematic review from African most populous country, the prevalence of high blood pressure or hypertension ranged between one-tenth to approximately half of the total population [6, 10, 14]. Notice that we may be tempted to think that the prevalence of high blood pressure among women of reproductive age in Benin is relatively low, when compared with some other SSA countries, however, previous studies from Benin reported less than half of this current prevalence [22, 23]. The upward trend in the prevalence of high blood pressure or hypertension, is consistent with a previous report where the overall pooled prevalence of hypertension in Africa was 19.7% in 1990, 27.4% in 2000 and 30.8% in 2010 [24]. Thus, suggest that the prevalence of hypertension is increasing in Africa, unfortunately, many hypertensive individuals may not be aware of their condition.

Based on the findings, older women had increased likelihood of high blood pressure. This is consistent with the findings from previous studies [6, 23]. The increased odds of aged women having high blood pressure, could be due to the biological effect of increased arterial resistance as a result of arterial thickening as individuals grow older. More so, the reduction in the odds of high blood pressure by women from highest neighbourhood socioeconomic disadvantaged level and the enlightened women been more likely to report high blood pressure [22], revealed epidemiological transition in resource-poor settings are usually explained by economic development leading to urbanization with increased sedentary lifestyle, deviations in dietary habits and other lifestyles. Benin is likely to be undergoing similar development. The well-off women had a higher prevalence of high blood pressure in this study. However, this may be due to the fact that advantaged women reported high blood pressure or had higher detection rate for hypertension because richer people have better access to medical service.

Furthermore, women’s geographical region and increased number of lifetime sex partners were associated with increased odds of high blood pressure. This is consistent with reports from a previous study [25]. A possible explanation is that women who suffer from intimate partner violence may have had higher lifetime number of sex partners [26, 27]. In previous studies, intimate partner violence was associated with hypertension [28, 29]. The differentials in high blood pressure by geographical location could be due to certain factors prevalent in such locations. It is possible that people who live in the same natural settlements have common feeding pattern, lifestyles, occupation, beliefs and practices that could determine their health conditions. Such factors could have been responsible for the variations in the odds of high blood pressure among women across diverse geographical locations in Benin.

### Strengths and limitations

Here, we used nationally representative data and the findings are generalizable for the women of reproductive age in Benin, West Africa. However, only an association of the factors and not causation can be inferred due to the cross-sectional nature of the data. Also, we were unable to explore other contributory risk factors of high blood pressure; such as overweight/obesity, salt intake, psychosocial stress, and other endogenous factors. In addition, high blood pressure was based on women’s self-report which lacks objective assessment.

## Conclusions

This study explored the prevalence and determinants of high blood pressure. In presenting an evidence-based context for government and other health policymakers on the strategies to reduce the occurrence of high blood pressure in Benin, detailed up-to-date information on the prevalence and factors of the condition has been provided in order to match this with appropriate interventions. The findings also suggest involvement of multi-dimensional strategies that will require interventions at individual and population levels. Health policymakers and government need to focus on widespread prevention and control interventions of hypertension through community health programmes. A notable intervention would be to adopt an approach to reduce risk distribution by directing resources to high-risk women. Integrating equity elements to interventions is also a key measure towards ensuring that policies and programmes aid high-risk women. Essentially, policymakers and stakeholders in the health sector need to institute nationwide population-based strategies towards creating awareness and educating people on the risk factors. It is hoped that the findings of this study will prompt appropriate policy response at country level towards improved detection, control and overall management of high blood pressure in Benin.

## Data Availability

Data for this study were sourced from Demographic and Health surveys (DHS) and available here: https://www.dhsprogram.com/data/available-datasets.cfm
